# Causal relationship between plasma lipidome and four types of pancreatitis: a bidirectional Mendelian randomization study

**DOI:** 10.3389/fendo.2024.1415474

**Published:** 2024-09-30

**Authors:** Runzhou Ma, Chengming Chen, Ziyi Wang, Huaibin Guo, Wanxing Zhang

**Affiliations:** ^1^ Department of Hepatobiliary Surgery, Hebei General Hospital, Shijiazhuang, China; ^2^ Department of Ophthalmology, Tangdu Hospital, The Air Force Military Medical University, Xi’an, China

**Keywords:** lipidome, pancreatitis, causal inference, bidirectional Mendelian randomization, sensitivity

## Abstract

**Background:**

Pancreatitis is a serious and complex inflammatory disease that imposes a severe effect on quality of life. Links between plasma lipidome and pancreatitis have been reported, some of which have not yet been clearly elucidated.

**Methods:**

Therefore, our study aimed to investigate the causal relationships between plasma lipidome and four types of pancreatitis by conducting a bidirectional, two-sample Mendelian randomization (MR) analysis. We obtained genetic variants associated with 179 lipid species from a Genome-wide association analysis of plasma lipidome. The aggregated statistical data of acute pancreatitis (AP), alcohol-induced acute pancreatitis (AAP), chronic pancreatitis (CP), and alcohol-induced chronic pancreatitis (ACP) from the FinnGen consortium were exploited as the outcome. The inverse variance weighted (IVW) technique as the main method was used for MR analysis and sensitivity analyses were used to evaluate heterogeneity and pleiotropy.

**Results:**

After FDR correction, SE (27:1/20:4) (OR = 0.938, 95%CI = 0.906-0.972, P = 4.38 × 10^-4^, PFDR = 0.039) was identified to be significantly associated with AP risk. Eight lipid species were identified to be significantly associated with CP risk: SE (27:1/20:4) (OR = 0.911, 95%CI = 0.869-0.954, P = 8.89 × 10^-5^, PFDR = 0.016), LPC (20:4) (OR = 0.892, 95%CI = 0.843-0.945, P = 9.74 × 10^-5^, PFDR = 0.009), PC (16:0_22:5) (OR = 0.880, 95%CI = 0.818-0.947, P = 6.29 × 10^-4^, PFDR = 0.028), PC (17:0_20:4) (OR = 0.893, 95%CI = 0.842-0.948, P = 1.76 × 10^-4^, PFDR = 0.010), PC (18:0_20:4) (OR = 0.920, 95%CI = 0.874-0.969, P = 1.70 × 10^-3^, PFDR = 0.038), PC (O-16:0/20:4) (OR = 0.871, 95%CI = 0.804-0.943, P = 6.95 × 10^-4^, PFDR = 0.025), PC (O-16:1/20:4) (OR = 0.890, 95%CI = 0.832-0.953, P = 7.85 × 10^-4^, PFDR = 0.023), and PE (O-18:1/20:4) (OR = 0.866, 95%CI = 0.791-0.947, P = 1.61 × 10^-3^, PFDR = 0.041). Furthermore, genetically predicted increased LPC (20:4) (OR = 0.862, 95%CI = 0.796-0.934, P = 3.00 × 10^-4^, PFDR = 0.027) and SM (34:2;O2) (OR = 0.753, 95%CI = 0.659-0.860, P = 2.97 × 10^-5^, PFDR = 0.005) levels were associated with decreased risk of ACP.

**Conclusions:**

Our findings provide evidence of causal associations between the specific types of lipidome and pancreatitis, offering new insights into future clinical research.

## 1 Introduction

Pancreatitis is an inflammatory disease characterized by severe abdominal pain and pancreatic exocrine and/or endocrine dysfunction, including acute pancreatitis (AP), acute recurrent pancreatitis and chronic pancreatitis (CP) (1). AP has become one of the most common gastrointestinal diseases requiring hospitalization, and its incidence continues to increase worldwide. Most AP is self-limiting, partly because repeated inflammation may progress to severe AP or even CP (2). Gallstone migration and alcohol abuse are the main causes of AP and CP (3). Recent study has shown that 43.5% of patients with alcoholic pancreatitis are readmitted due to recurrent pancreatitis, which is much greater than the 22.1% of patients with non-alcoholic pancreatitis. In addition, alcoholic acute pancreatitis (AAP) is more likely to progress to CP than non-alcoholic pancreatitis (4, 5). It is generally accepted that plasma lipid levels are also associated with the risk of pancreatitis. A systematic review has shown that hypertriglyceridemia has become a common cause of AP following alcohol as well as gallstone disease, accounting for 10% of all pancreatitis episodes (6). Plasma lipids are usually measured via high-density lipoprotein cholesterol (HDL-C), low-density lipoprotein cholesterol (LDL-C), triglycerides (TG), and total cholesterol (TC) (7). In recent years, advances in modern efficient lipidomics techniques have expanded our understanding of the width of circulating lipids. The plasma lipidome encompasses a comprehensive profile of lipids present in the plasma, critically contributing to energy storage, cellular membrane formation, and intercellular communication. Its significance in diagnosing and managing a variety of health conditions, as well as its role in uncovering the underlying biochemical mechanisms, has been increasingly recognized (8). A more detailed classification of circulating lipids, including glycerolipids, glycerophospholipids, sphingolipids, and sterols, presumably ameliorates the risk and severity estimation of pancreatitis compared with the traditional four lipid panels.

The advent of Genome-wide association studies (GWAS) techniques and high-throughput technologies has advanced our understanding of the complex genetic factors behind diseases (9). Mendelian randomization (MR) analysis is an approach using genetic variants as instrumental variables (IVs) for exposure to investigate the causal relationship between the exposure and outcome. Compared with traditional observational studies, it has the advantage of overcoming the problems of confounding and reverse causality (10). Previous MR studies have reported that triglyceride levels and fatty acid unsaturated levels have a causal effect on pancreatitis risk (11). Yet, there is still a dearth of systematic evaluation of a broader range of plasma lipidome to pancreatitis predisposition.

In this study, we performed a large-scale bidirectional two-sample MR analysis to explore and establish protective causality for different types of plasma lipidome in the diversity of pancreatitis. Further understanding of the relationship between these plasma lipidome and the pathophysiology of pancreatitis will provide a new perspective for the diagnosis and treatment of pancreatitis.

## 2 Materials and methods

### 2.1 Study design

**Figure 1:**
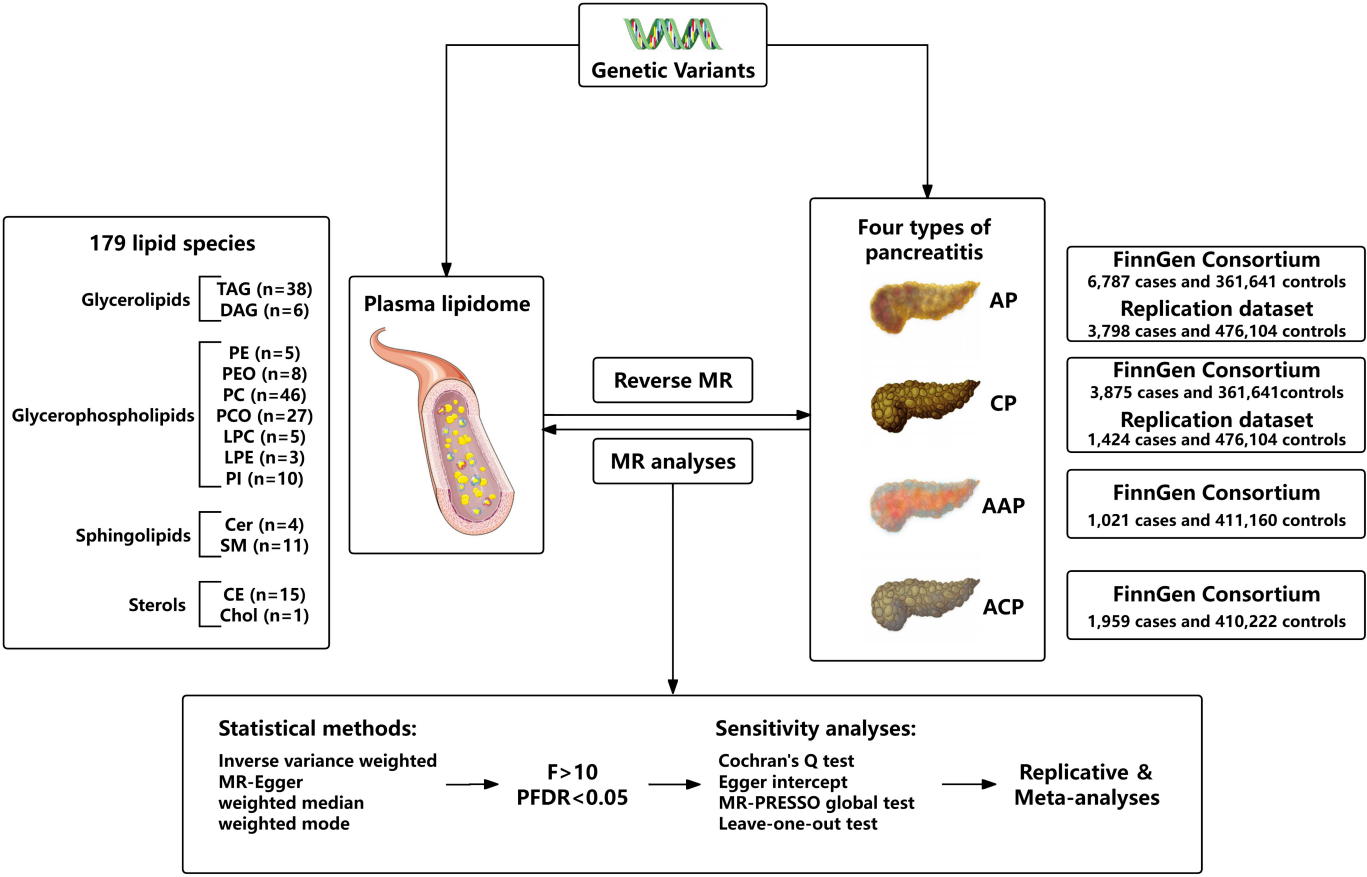
Flow chart of this study.

We assessed the bidirectional causal relationship between 179 lipid levels and four types of pancreatitis based on a two-sample MR approach. To perform MR analysis, three core assumptions must be followed by IVs: Firstly, there is a strong correlation between exposure and genetic variants; Secondly, there should be no association between genetic variants and potential confounding factors; Thirdly, genetic variants can only affect outcome through exposure. To ensure that MR analysis meets the three core assumptions. The overall research design is depicted in **Figure 1**.

### 2.2 Data sources

For the exposure instrument, we used the summary statistics from the recent large-scale GWAS on plasma lipidome, which are publicly available from the GWAS Catalog (https://www.ebi.ac.uk/gwas/, accession numbers from GCST90277238 to GCST90277416) for each lipid species. This GWAS analysis was performed based on 7174 Finnish individuals to test around 13 million variants. By using shotgun lipidomics, 179 lipid species were divided into four categories, including glycerolipids (GL) (n = 44), glycerophospholipids (GP) (n = 104), sphingolipids (SL) (n = 15), and sterols (ST) (n = 16). Specifically, GL included triacylglycerol (TAG) and diacylglycerol (DAG), GP included lysophosphatidylcholine (LPC), lysophosphatidylethanolamine (LPE), phosphatidylcholine (PC), phosphatidylcholine-ether (PCO), phosphatidylethanolamine (PE), phosphatidylethanolamine-ether (PEO), phosphatidylinositol (PI), SL included ceramide (Cer) and sphingomyelin (SM), and ST included cholesterol ester (CE)/sterol ester (SE) and cholesterol (Chol) (**Supplementary Table 1**). The lipid species are named in the following notation: class name (sum of carbon atoms: sum of double bonds; sum of hydroxyl groups). The annotation of lipid subspecies includes information on their acyl moieties and, if available, on their sn-position. The acyl chains are separated either by “_” if the sn-position on the glycerol cannot be resolved or else by “/” (12).

GWAS summary data for pancreatitis were derived from the FinnGen consortium (https://r10.finngen.fi/). The latest R10 release of the FinnGen consortium data was used, which contains 6,787 cases and 361,641 controls for AP (K11_ACUTPANC), 3,875 cases and 361,641controls for CP (K11_CHRONPANC), 1,021 cases and 411,160 controls for AAP (ALCOPANCACU), 1,959 cases and 410,222 controls for ACP (ALCOPANCCHRON). The replication analyses for pancreatitis utilized GWAS data by Sakaue et al. from the IEU Open GWAS database (https://gwas.mrcieu.ac.uk/). For AP (ebi-a-GCST90018789), there were 3,798 cases and 476,104 controls of European individuals. For CP (ebi-a-GCST90018821), there were 1,424 cases and 476,104 controls of European individuals (13).

### 2.3 Selection of instrumental variables

Only a small amount of SNPs were chosen as IV at the strict threshold (P < 5 × 10^-8^). According to recent research, we use the more inclusive threshold (P < 1 × 10^-5^) to obtain more IVs. To ensure the independence of each IV, SNPs within the genomic window of 10,000 endonuclides were pruned, and a threshold of r^2^ = 0.01 was applied to mitigate the effects of linkage disequilibrium (LD). Then, remove the palindromic SNPs and duplicate SNPs from IV. In addition, to avoid bias from weak instrumental variables, we used the F-statistic to assess the statistical strength of the association between each SNP and exposure. F > 10 was generally considered a threshold for powerful IVs. Typically, IVs with low F statistics (< 10) were removed from our analysis (14, 15). In reverse MR analysis, to ensure sufficient SNPs for alcohol-related pancreatitis, we selected a genome-wide significance level (5 × 10^-6^) to identify potential causality.

### 2.4 Statistical analysis

The random-effect inverse-variance weighted (IVW) method was used as the main MR approach, which can obtain the most accurate and reliable causal effect between plasma lipidome and pancreatitis (16). For exposures containing one or two SNPs, IVW with fixed effects was used. Multiplicative random effects were used for MR analysis with more than three SNPs or heterogeneity in the data. Other methods including MR-Egger and weighted median were also used as additional analysis (17, 18). Through the integration of multiple MR methods to improve the accuracy and stability of estimating. For statistical analysis, Cochran’s Q test (P < 0.05) was performed to test the heterogeneity and the MR-Egger regression intercept (P < 0.05) was used to identify horizontal pleiotropy (19). Meanwhile, MR-PRESSO was used to detect the presence of SNP outliers and corrected estimates by removing outliers if necessary. Leave-one-out analysis was performed to exclude the outlier variants one by one and evaluate the degree of dependence of the results on a specific variant, further excluding possible horizontal pleiotropy (20). To avoid increased Type 1 errors from multiple hypothesis testing, we corrected the significance threshold using the false discovery rate (FDR) correction (21). Only results with FDR-corrected P-values less than 0.05 were included in the final analysis. Finally, we repeated the MR analyses with the other two pancreatitis GWAS summary datasets and conducted meta-analyses to verify the reliability of the results. All MR analyses were done via the TwoSampleMR package (version 0.5.8) in the R (4.3.2) software, and meta-analyses were performed in the R-based “meta” package.

## 3 Results

### 3.1 Exploring the impact of plasma lipidome on the causal relationship of AP

**Figure 2:**
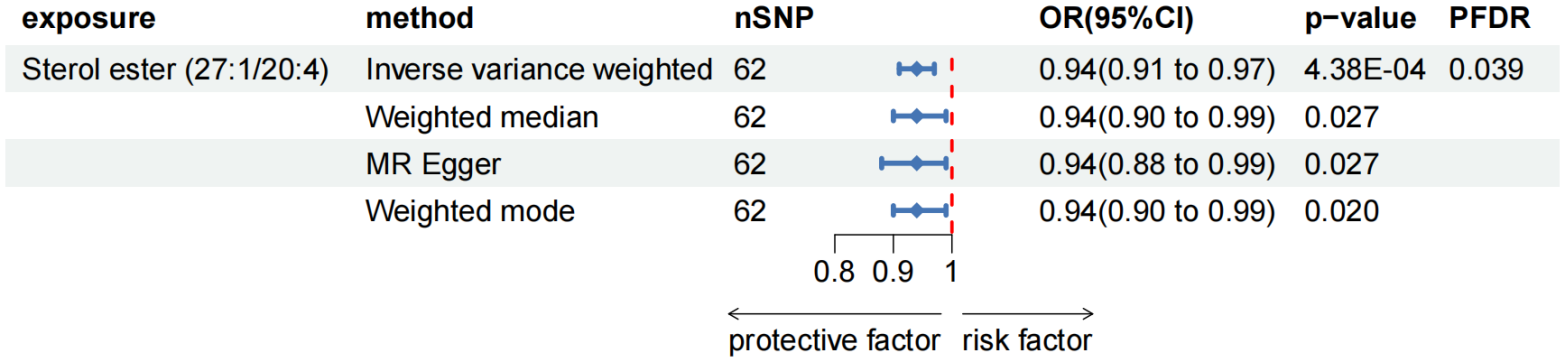
Forest plot for the causal effect of circulating immune cells on the risk of AP using different methods; nSNP, number of single nucleotide polymorphisms; OR, odds ratio; CI, confidence interval.

As the primary analysis methods, we employed the two-sample MR analysis and IVW approach to look into the causal association between plasma lipidome and AP. After FDR test adjustment (PFDR < 0.05), SE (27:1/20:4) was found to have a protective causal relationship to AP: (OR = 0.938, 95%CI = 0.906-0.972, P = 4.38 × 10^-4^, PFDR = 0.039). Results from other MR methods were similar: MR-Egger (OR = 0.936, 95%CI = 0.884-0.991, P = 0.027), weighted median (OR = 0.944, 95%CI = 0.898-0.994, P = 0.027), and weighted mode (OR = 0.944, 95%CI = 0.900-0.990, P = 0.020) (**Figure 2**; **Supplementary Table 2**). The results of sensitivity analysis and the leave-one-out plot are detailed in the Supplementary Materials (**Supplementary Table 2**; **Supplementary Figure 1**). Moreover, replication analyses and meta-analyses have further bolstered the credibility of these results (**Supplementary Table 2**). In the reverse MR results of plasma lipidome and AP, all MR analysis p-values are greater than 0.05 after FDR test adjustment (PFDR > 0.05), indicating that AP does not affect the included lipidome (**Supplementary Table 2**).

### 3.2 Exploring the impact of plasma lipidome on the causal relationship of CP

**Figure 3:**
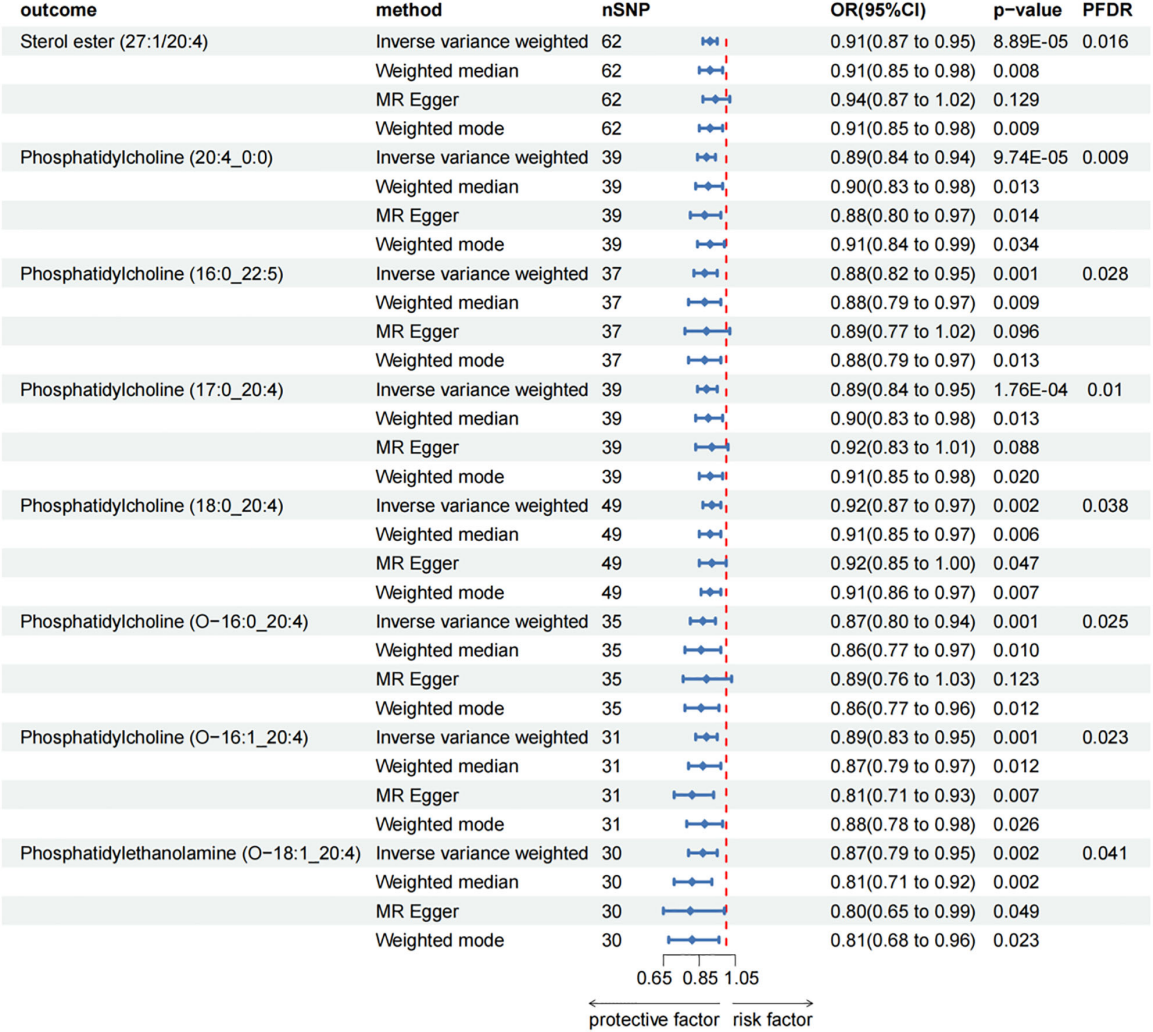
Forest plot for the causal effect of circulating immune cells on the risk of CP using different methods; nSNP, number of single nucleotide polymorphisms; OR, odds ratio; CI, confidence interval.

After FDR adjustment (PFDR < 0.05), we detected protective effects of eight lipid levels on CP: one in the CE class, one in the LPC class, three in the PC class, two in the PCO class, and one in the PEO class. The IVW analysis results for all plasma lipidome were: SE (27:1/20:4) (OR = 0.911, 95%CI = 0.869-0.954, P = 8.89 × 10^-5^, PFDR = 0.016), LPC (20:4) (OR = 0.892, 95%CI = 0.843-0.945, P = 9.74 × 10^-5^, PFDR = 0.009), PC (16:0_22:5) (OR = 0.880, 95%CI = 0.818-0.947, P = 6.29 × 10^-4^, PFDR = 0.028), PC (17:0_20:4) (OR = 0.893, 95%CI = 0.842-0.948, P = 1.76 × 10^-4^, PFDR = 0.010), PC (18:0_20:4) (OR = 0.920, 95%CI = 0.874-0.969, P = 1.70 × 10^-3^, PFDR = 0.038), PC (O-16:0/20:4) (OR = 0.871, 95%CI = 0.804-0.943, P = 6.95 × 10^-4^, PFDR = 0.025), PC (O-16:1/20:4) (OR = 0.890, 95%CI = 0.832-0.953, P = 7.85 × 10^-4^, PFDR = 0.023), PE (O-18:1/20:4) (OR = 0.866, 95%CI = 0.791-0.947, P = 1.61 × 10^-3^, PFDR = 0.041). Furthermore, the MR-Egger methods of four of them were: SE (27:1/20:4) (OR = 0.943, 95%CI = 0.875-1.016, P = 0.129), PC (16:0_22:5) (OR = 0.887, 95%CI = 0.773-1.018, P = 0.096), PC (17:0_20:4) (OR = 0.915, 95%CI = 0.829-1.011, P = 0.088) and PC (O-16:0/20:4) (OR = 0.887, 95%CI = 0.764-1.029, P = 0.123). Although P > 0.05 of MR-Egger in these results, the beta values of the four MR analysis methods were all < 0, indicating that they were all in the same direction (**Figure 3**; **Supplementary Table 3**). The biggest difference between the MR-Egger method and IVW is that the MR-Egger method considers the existence of intercept term. In the absence of heterogeneity and horizontal pleiotropy, the IVW method provides a more accurate estimate than the MR-Egger method. We will give priority to the results of IVW. Therefore, above all, the results of MR analysis were still statistically significant. The results of the auxiliary analysis of the remaining four types were consistent with the observed results. All sensitivity analyses confirmed the robustness of the observed causal relationship, and further validation through subsequent replication studies and meta-analyses provided additional support for these results (**Supplementary Table 2**). In the reverse MR results of lipidome and CP, all MR analysis p-values are greater than 0.05 after FDR test adjustment (PFDR > 0.05), indicating that CP has no effect on the included lipidome. All results are detailed in the Supplementary Materials (**Supplementary Table 3**; **Supplementary Figure 2**).

### 3.3 Exploring the impact of plasma lipidome on the causal relationship of AAP

Based on the IVW test estimates, no lipid level was identified at a significance of 0.05. In the reverse MR results of lipidome and AAP, the p-value of seven plasma lipid levels was less than 0.05. However, after adjusting for FDR testing, all MR analysis p-values were greater than 0.05 after FDR test adjustment (PFDR > 0.05), indicating that AAP has no effect on the included lipidome. In summary, we did not find a causal relationship between plasma lipidome and AAP. The results are shown in the Supplementary Materials (**Supplementary Table 4**).

### 3.4 Exploring the impact of plasma lipidome on the causal relationship of ACP

**Figure 4:**
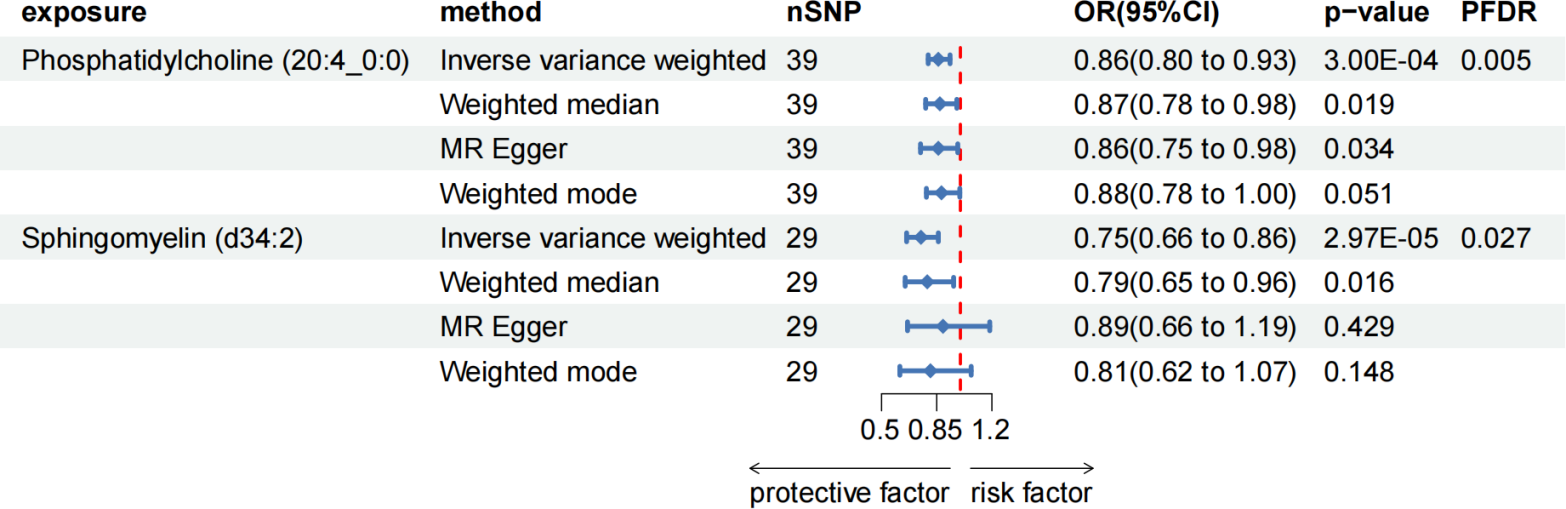
Forest plot for the causal effect of circulating immune cells on the risk of ACP using different methods; nSNP, number of single nucleotide polymorphisms; OR, odds ratio; CI, confidence interval.

After FDR adjustment (PFDR < 0.05), we detected protective effects of LPC (20:4) and SM (34:2;O2) levels on ACP. Among them, the odds ratio (OR) of LPC (20:4) on ACP risk estimated by IVW method was 0.862 (95%CI = 0.796-0.934, P = 3.00 × 10^-4^, PFDR = 0.027). Results from other MR methods were similar: MR-Egger (OR = 0.857, 95%CI = 0.747-0.983, P = 0.034), weighted median (OR = 0.873, 95%CI = 0.780-0.977, P = 0.019), and weighted mode (OR = 0.884, 95%CI = 0.784-0.996, P = 0.051). The ratio of SM (34:2;O2) levels to ACP risk (OR) was assessed with the IVW method and was 0.753 (95%CI = 0.659-0.860, P = 2.97 × 10^-5^, PFDR = 0.005). Results from other MR methods were similar: MR-Egger (OR = 0.888, 95%CI = 0.665-1.186, P = 0.428), weighted median (OR = 0.789, 95%CI = 0.650-0.957, P = 0.016), and weighted mode (OR = 0.811, 95%CI = 0.616-1.068, P = 0.148) (**Figure 4**; **Supplementary Table 4**). In the reverse MR results of lipidome and ACP, all MR analysis p-values were greater than 0.05 after FDR test adjustment (PFDR > 0.05), indicating that ACP had no effect on the included lipidome (**Supplementary Table 4**).

**Figure 5:**
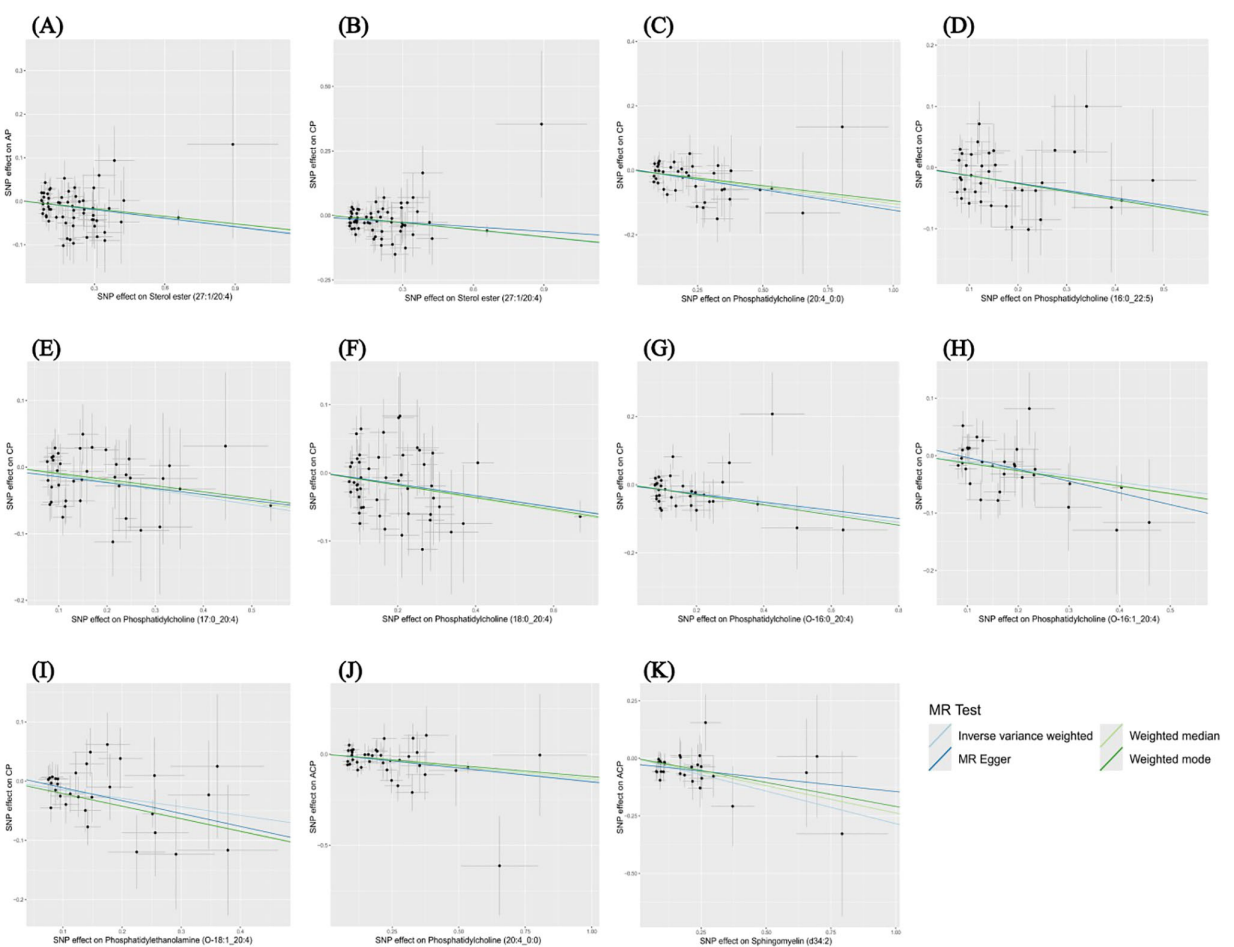
Scatter plots depicting the causal relationship between the effect sizes of SNPs on plasma lipidome components (x-axis) and the corresponding effect sizes for pancreatitis (y-axis). Each plot focuses on a specific lipid species: **(A)** Sterol ester (27:1/20:4) on AP. **(B)** Sterol ester (27:1/20:4) on CP. **(C)** Phosphatidylcholine (20:4_0:0) on CP. **(D)** Phosphatidylcholine (16:0_22:5) on CP. **(E)** Phosphatidylcholine (17:0_20:4) on CP. **(F)** Phosphatidylcholine (18:0_20:4) on CP. **(G)** Phosphatidylcholine (O-16:0_20:4) on CP. **(H)** Phosphatidylcholine (O-16:1_20:4) on CP. **(I)** Phosphatidylethanolamine (O-18:1_20:4) on CP. **(J)** Phosphatidylcholine (20:4_0:0) on ACP. **(K)** Sphingomyelin (d34:2) on ACP.

The results of the other three methods and sensitivity analyses confirmed the stability of the observed causal associations. Specifically, the Cochran’s Q test, MR-Egger intercept and MR-PRESSO test were examined to rule out the heterogeneity and horizontal pleiotropy (**Supplementary Table 4**). In addition, leave-one-out plots showed the stability of the results (**Supplementary Figure 3**). All scatter plots regarding the causal relationship between plasma lipidome and the risk of pancreatitis can be found in **Figure 5**.

## 4 Discussion

Based on a large amount of publicly available genetic data, our study explored the causal relationships between 179 plasma lipidome and four types of pancreatitis. In this study, we discovered potential causal relationships between one type of plasma lipid level and AP, eight different types of plasma lipid levels and CP, and two types of plasma lipid levels and ACP.

Our study found that the risk of AP and CP decreased with an increase of SE (27:1/20:4), a cholesterol ester belonging to the CE (20:4) species. The synthesis and hydrolysis of CEs play important roles in the maintenance of cholesterol homeostasis. CEs are formed by the esterification of cholesterol and fatty acids, which is the main form of cholesterol storage and transport. The free cholesterol in plasma is catalyzed by lecithin cholesterol acyltransferase (LCAT) to accept the fatty acyl group on the lecithin molecule to form cholesterol ester and lysed lecithin (22). The newly formed CEs are subsequently incorporated into the hydrophobic core of HDL, forming a concentration gradient on the free cholesterol content between its surface and plasma and surrounding cells, which is conducive to driving the net outflow or removal of cholesterol from the cells (23, 24). Limited research has been conducted on the role of plasma CEs in pancreatitis as of yet. The mechanism may be related to the maintenance of cholesterol metabolic homeostasis and participation in reverse cholesterol transport to reduce cholesterol accumulation, thus inhibiting inflammation and alleviating pancreatic toxicity (25).

Glycerophospholipids not only constitute biofilms but also regulate a variety of biological processes including cell proliferation, apoptosis, immunity, angiogenesis and inflammation through related pathways (26). According to research, glycerophospholipid metabolic pathway was identified as the key metabolic pathway of CP (27). In this study, phosphatidylcholine (20:4_0:0), which belongs to lysophosphatidylcholine (LPC), has been proven to be significantly associated with a reduced risk of CP and ACP. During CP development, apoptotic cells induce M2a macrophages to exert an anti-inflammatory response and relieve fibrosis by releasing LPC as a “find me” signal (28). Besides, the acyl chain length and degree of saturation of LPC lead to its different roles in the development of inflammatory diseases. Saturated LPC (16:0) is a potential inflammatory mediator that induces the release of pro-inflammatory cytokines. Polyunsaturated acyl LPC (20:4) acts as an anti-inflammatory lipid mediator, inhibiting inflammation caused by saturated LPC. Its anti-inflammatory effect is related to reducing plasma leakage and inflammatory cell activation, inhibiting the production of inflammatory mediators (IL-6, NO, PGE, etc.), and increasing anti-inflammatory factors (IL-10 and IL-4) (29). The result is consistent with our findings of this study.

According to this study, the risk of CP decreased as the three kinds of PC increased: (16:0_22:5), (17:0_20:4), and (18:0_20:4). Phosphatidylcholine (PC) is the predominant phospholipid found in cell membranes and has been linked to inflammatory processes by shaping membrane composition and fluidity (30). Previous studies have shown that PC is associated with improved insulin resistance and abnormal lipid accumulation and inhibits the synthesis and release of multiple inflammatory factors, such as IL-1β and TNF-α (31, 32). PC metabolites can also inhibit endoplasmic reticulum stress and subsequent oxidative stress response, maintain Th17/Treg cell immune balance, and improve tissue inflammation (33). Serum metabolomic studies have shown that PC metabolites can be used to diagnose CP and are associated with pancreatic exocrine insufficiency in CP (27, 34).

Ether lipids are a unique class of glycerophospholipids that have an alkyl chain connected to the sn-1 position by an ether bond. They are initially synthesized in peroxisomes and then processed into PCO and PEO in the endoplasmic reticulum (ER) (35). Ether lipids regulate cell signaling and act as antioxidants, mediating the relationship between oxidative stress and inflammation. The Daniel Hornburg team found that PEOs were significantly associated with health phenotypes, including low SSPG levels and high HDL levels (36). And we found that three plasma ether lipids (Phosphatidylcholine (O-16:0_20:4), Phosphatidylcholine (O-16:1_20:4) and Phosphatidylethanolamine (O-18:1_20:4))could reduce the risk of CP.

The synthesis of SM begins with ER, goes through a series of enzymatic reactions in the Golgi apparatus and plasma membrane, and is finally synthesized by the substrate ceramide and PC. Sphingomyelin has a similar structure to glycerol phospholipids, which gives it similar properties and functions (37). Like glycerophospholipid metabolism, sphingolipid metabolism is also thought to be related to CP. However, research on the role of sphingomyelin in ACP is limited. Recent studies have shown differential expression of sphingomyelin in the plasma of patients with CP and pancreatic cancer (38). Additionally, sphingosine 1-phosphate (S1P), a metabolite of sphingomyelin, is associated with the severity of AP (39). According to this study, we discovered a correlation between sphingomyelin (d34:2) levels and a decreased risk of ACP.

This study conducted a two-sample MR analysis based on large published GWAS datasets and performed causal inferences through a variety of powerful MR analysis techniques. The results were robust and not influenced by horizontal pleiotropy or other factors. Additionally, to control for false positive results in multiple hypothesis tests, we used FDR to control for statistical bias caused by multiple comparisons. However, there are some limitations to the study. First, the GWAS data used in our study was limited to European populations, limiting the generalizability of the findings to other ethnic groups. Second, due to the lack of individual information, we are unable to conduct further stratified analysis of the population (e.g., sex, age, etc.). Third, although multiple sensitivity analyses have been performed to evaluate the hypotheses of MR studies, it is not possible to completely rule out confounding bias or horizontal pleiotropy. Finally, we used a looser threshold to evaluate the results, which may increase some false positives despite FDR correction. However, the strong association between plasma lipidome and pancreatitis can be evaluated more comprehensively.

## 5 Conclusion

Overall, we have demonstrated the causal associations between plasma lipidome and four types of pancreatitis through comprehensive bidirectional MR analyses. Besides, we highlighted the different structures of lipids and their potential differential effects across various molecular subtypes. Our findings may provide new avenues for researchers to explore the biological mechanisms of pancreatitis and help explore early intervention and treatment. The pathogenesis of pancreatitis is very intricate, and the unique biological effects of lipid subclasses beyond the traditional lipid spectrum on pancreatitis need to be further studied.

## Data Availability

The original contributions presented in the study are included in the article/**Supplementary Material**. Further inquiries can be directed to the corresponding author/s.
