# Supplementary material for: Causal relationship between plasma lipidome and four types of pancreatitis: a bidirectional Mendelian randomization study
Source: Front Endocrinol (Lausanne). 2024 Sep 30;15:1415474. doi: 10.3389/fendo.2024.1415474 (PMC11471641; doi:10.3389/fendo.2024.1415474)
Supplement: Supplementary file 1 [file DataSheet1.pdf]

Forest plots for MR leave-one-out analysis between plasma lipidome of and pancreatitis risk. Within each panel, the black points represent the causal estimate after discarding each SNP in turn. Red points represent the pooled IVW estimates. Horizontal lines denote 95% confidence intervals.

Supplementary Figure 1
Supplementary Figure 2
Supplementary Figure 3

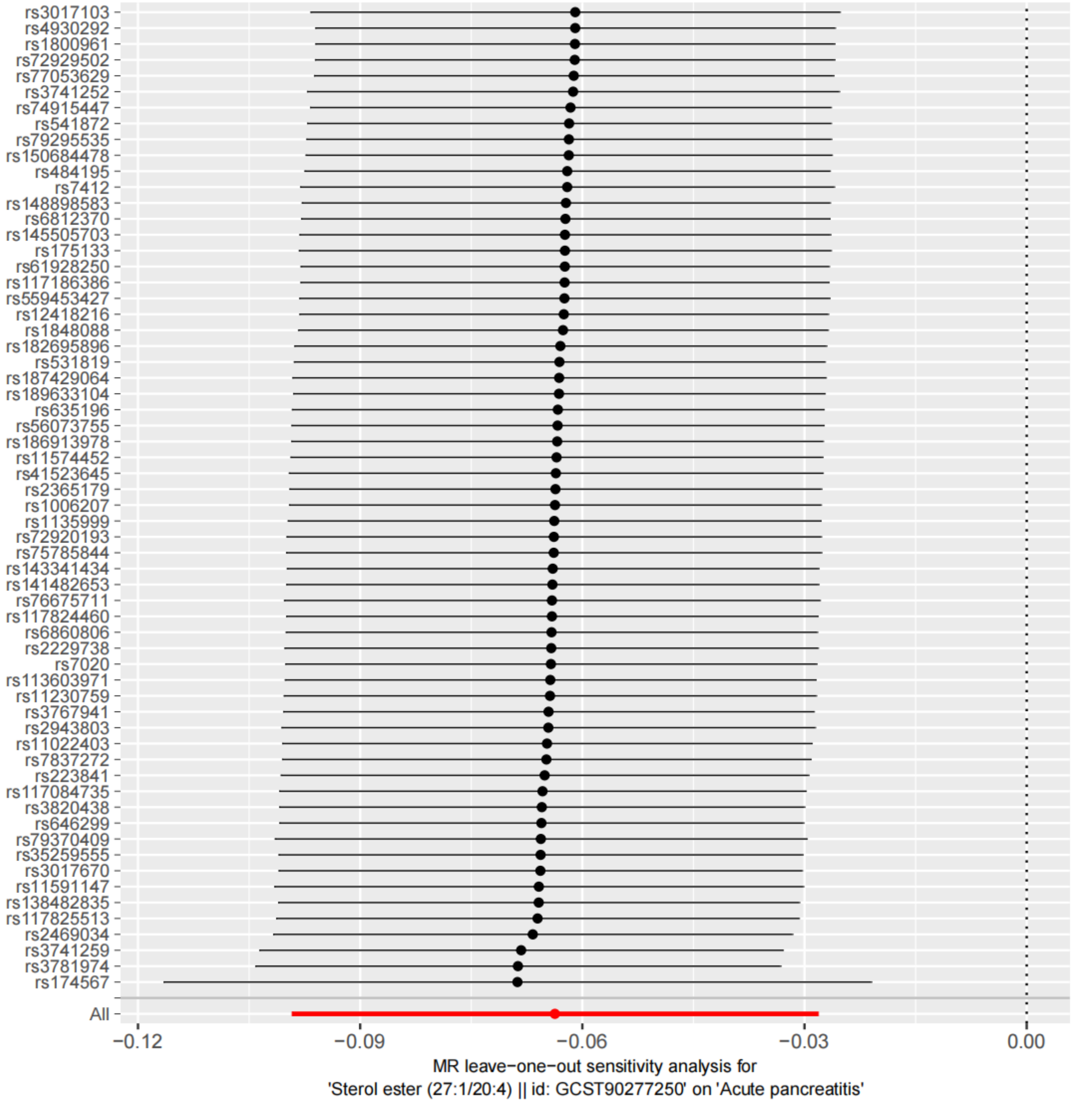


**Supplementary Figure 1.** The leave-one-out plot for "SE (27:1/20:4)" on "AP".

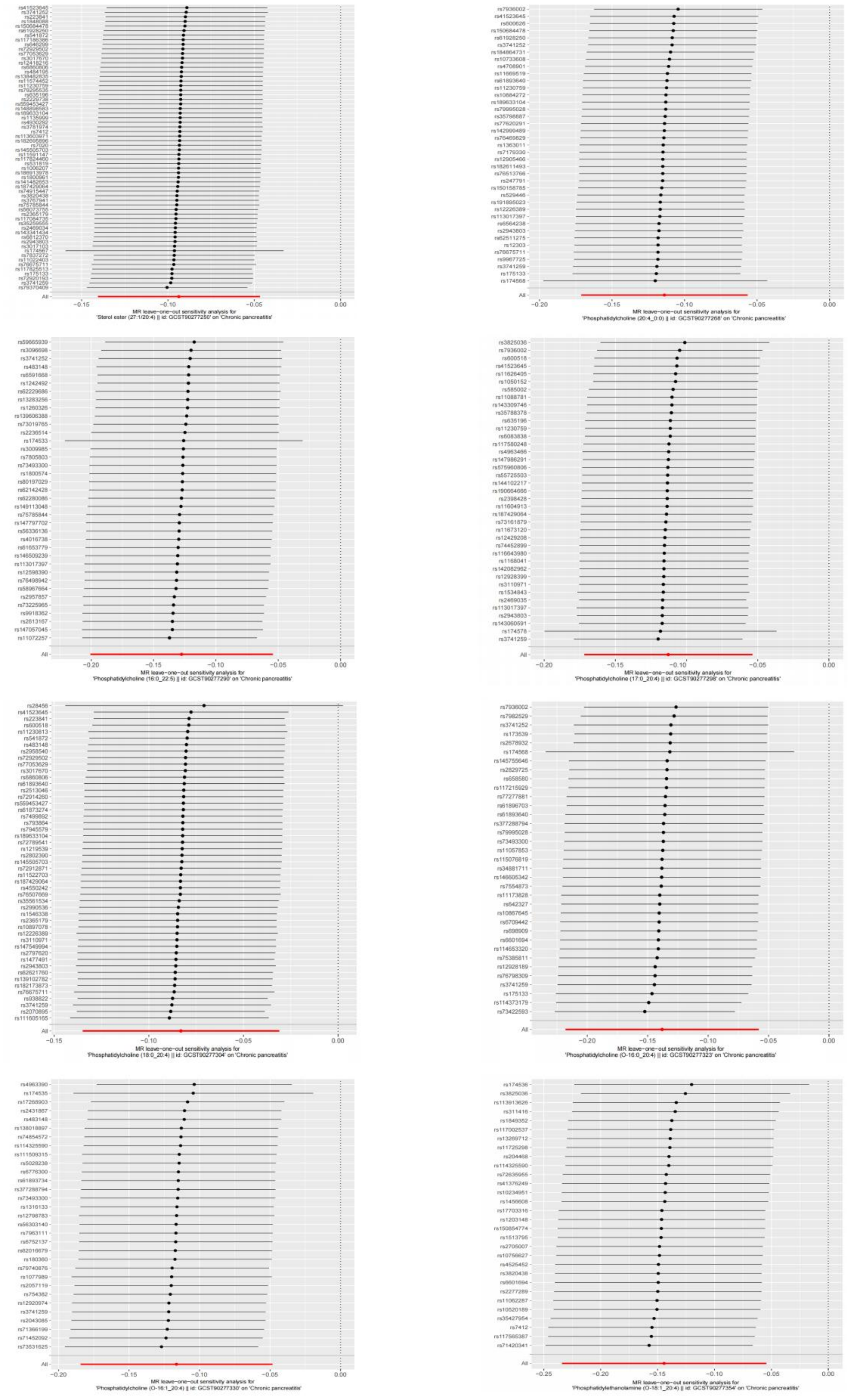


**Supplementary Figure 2.** The results of MR leave-one-out sensitivity analysis in CP.

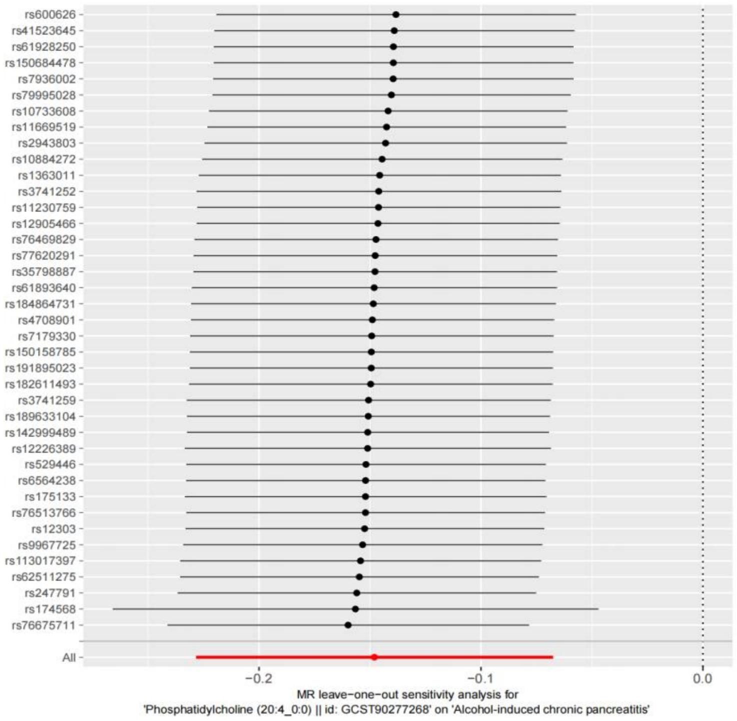


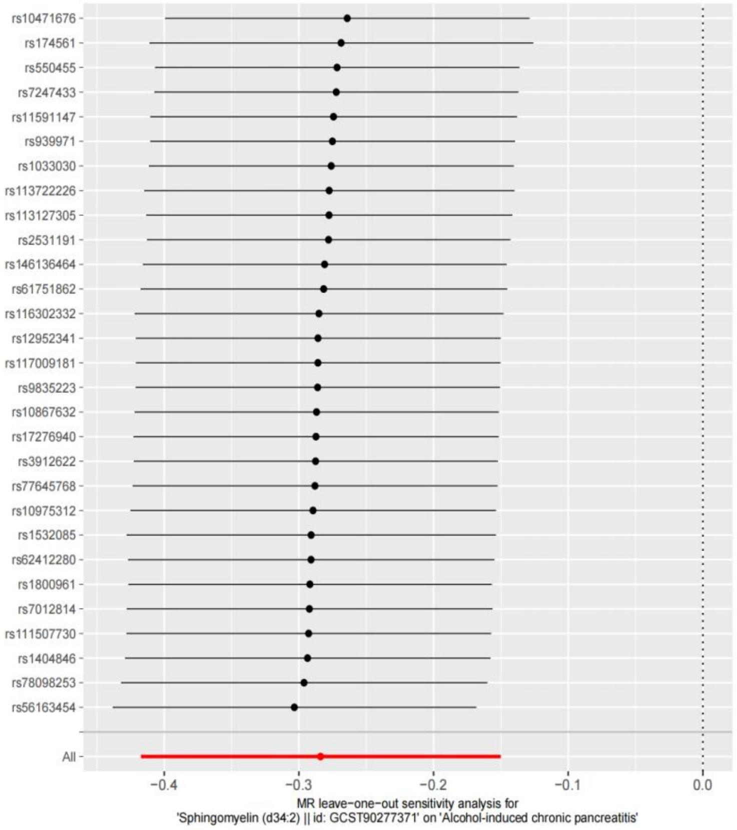


**Supplementary Figure 3.** The results of MR leave-one-out sensitivity analysis in ACP.
